# The American Rhinological Association

**Published:** 1888-07

**Authors:** 


THE AMERICAN RHINOLOGICAL ASSOICATION will hold its Sixth Annual Meeting at Cincinnati, Ohio, September, 12, 13 and 14, 1888. Dr. John North, Secretary, Keokuk, Iowa.

RECEIPTS will appear in our next.

MEMBERS OF THE PROFESSION who desire an engraving, accompanied by an autograph signature of the late Dr. C. R. Agnew, should send their names and addresses to Dr. Charles H. May, 640 Madison Avenue, New York City, at once. When all such names shall have been recorded, those who have requested a copy of the engraving will be notified of the cost of the same, either by the publisher, or by the committee having the matter in charge.

